# Younger postnatal age is associated with a lower heart rate on Holter monitoring during the first week of life

**DOI:** 10.1007/s00431-023-04914-4

**Published:** 2023-03-08

**Authors:** Asta Uusitalo, Antti Tikkakoski, Pieta Lehtinen, Kaisa Ylänen, Päivi H. Korhonen, Tuija Poutanen

**Affiliations:** 1 Department of Pediatrics, Tampere University Hospital, PO BOX 2000, FI-33521 Tampere, Finland; 2 Tampere Center for Child, Adolescent and Maternal Health Research, Faculty of Medicine and Health Technology, Tampere University, Tampere, Finland; 3 Department of Clinical Physiology and Nuclear Medicine, Tampere University Hospital, Tampere, Finland

**Keywords:** Bradycardia, Electrocardiography, Holter, Neonatal

## Abstract

To evaluate heart rate (HR), the presence of extrasystoles and other Holter findings among healthy newborns, and to collect data for new normal limits for Holter parameters in newborns. For this cross-sectional study, 70 healthy term newborns were recruited to undergo 24-h Holter monitoring. Linear regression analysis was used in HR analyses. The age-specific limits for HRs were calculated using linear regression analysis coefficients and residuals. The mean (SD) age of the infants was 6.4 (1.7) days during the recording. Each consecutive day of age raised the minimum and mean HR by 3.8 beats per minute (bpm) (95% CI: 2.4, 5.2; *P* < .001) and 4.0 bpm (95% CI: 2.8, 5.2; *P* < .001), respectively. Age did not correlate with maximum HR. The lowest calculated limit for minimum HR ranged from 56 bpm (aged 3 days) to 78 bpm (aged 9 days). A small number of atrial extrasystoles and ventricular extrasystoles were observed in 54 (77%) and 28 (40%) recordings, respectively. Short supraventricular or ventricular tachycardias were found in 6 newborns (9%).

*Conclusion*: The present study shows an increase of 20 bpm in both the minimum and mean HRs of healthy term newborns between the 3rd and 9th days of life. Daily reference values for HR could be adopted in the interpretation of HR monitoring results in newborns. A small number of extrasystoles are common in healthy newborns, and isolated short tachycardias may be normal in this age group.

**Table:** 

**What is Known:**
• *The current definition of bradycardia in newborns is 80 beats per minute.*
• *This definition does not fit into the modern clinical setting of continuously monitored newborns, where benign bradycardias are commonly observed.*
**What is New:**
• *A linear and clinically significant increase in heart rate was observed in infants between the ages of 3 and 9 days. *
• *It appears as though lower normal limits for heart rate could be applied to the youngest newborns.*

## Introduction

During the neonatal period, major changes—such as a decrease in pulmonary vascular resistance, closure of fetal shunts, and a decline in hemoglobin levels—occur in the cardiovascular system. In addition, a progressive maturation of cardiac autonomic nervous control occurs during early postnatal life [1]. The above-mentioned changes affect the heart rate (HR) of newborns.

The lower limit for normal HR in newborns is commonly considered to be 80 beats per minute (bpm) [2]. Recently, Bohnhorst et al. challenged this definition by reporting that 30% of healthy newborns have bradycardias below 80 bpm [3].

Continuous HR monitoring is becoming increasingly common among newborns admitted to neonatal units. Large studies have recently presented new reference values for electrocardiogram (ECG) parameters [4, 5]. However, standard ECGs depict only a brief period of the HR, and a true resting state ECG can be difficult to obtain, especially in infants. There is a need for new HR reference values based on long-term HR monitoring.

During the neonatal period, extrasystoles are primarily a benign, self-resolving phenomenon [6]. The number of healthy newborns with extrasystoles varies significantly between studies, and the abnormal amount of extrasystoles in newborns remains unknown. In addition, there are still gaps in the understanding of cardiac arrhythmias during infancy.

This study’s purpose was to utilize modern Holter analytics and evaluate HR and heart rhythm in healthy newborns during the first week of life. The aim was to collect data for new normal limits for Holter parameters in newborns.

## Materials and methods

**Fig. 1:**
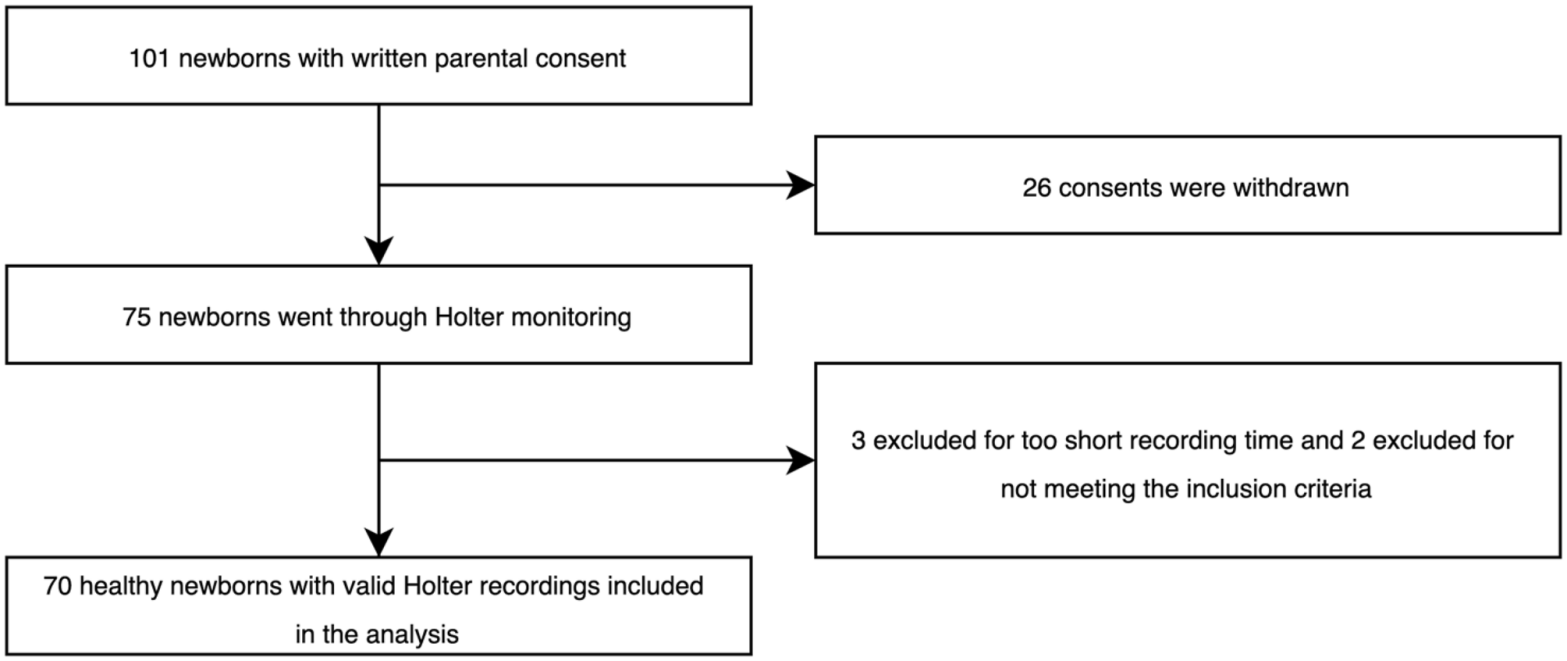
Flow diagram of study population selection

For this cross-sectional study, healthy term (≥ 37 + 0/7 weeks of gestation) newborns were recruited to undergo 24-h Holter monitoring (Fig. 1). The newborns were recruited after birth from the maternity wards of Tampere University Hospital, Tampere, Finland, between December 2018 and April 2021. The study complied with the Declaration of Helsinki, and the Regional Ethics Committee of Tampere University Hospital approved the study. Written informed consent was obtained from the parents.

The study’s exclusion criteria included maternal hypertension or gestational diabetes mellitus that was treated with medication, maternal smoking or substance abuse, regular maternal medication that potentially affected newborns’ HRs (e.g., betablockers, beta sympathomimetics, selective serotonin reuptake inhibitors, anticonvulsants, thyroid medication), or Apgar scores below 7, hypoglycemia (blood sugar levels below 2.6 mmol/l), heart disease, suspected or diagnosed infection of the newborn, or admission to the neonatal ward. Newborns with Holter recordings of less than 18 h were excluded.

All the newborns were clinically examined, and a 12-lead ECG was performed. Reference ranges by Davignon et al. were applied to the ECGs [7]. Echocardiography (echo) and additional Holter recordings were performed if clinically indicated. Demographic data were obtained from medical records. Birth size was evaluated according to Finnish growth curves [8].

Twenty-four-hour Holter monitoring was initiated at the outpatient clinic after discharge from the hospital. A diary of the symptoms and activities was completed during the recording. A SEER Light or SEER Light Extend Holter recorder (GE Medical Systems Information Technologies, Milwaukee, WI, USA) with three-channel recordings was used for all subjects. Analysis of the recorded Holter data was performed with MARS V8 software (GE Medical Systems Information Technologies, Milwaukee, WI, USA). The software automatically analyzed the HR, maximum R-R interval, number of QRS complexes, and number and runs of atrial premature contractions (APCs) and ventricular premature contractions (VPCs). The software calculated HR in bpm from 10 R-R intervals. After automated analysis, Holter data were checked for any misinterpretation of the software and edited manually by trained nurses. The final Holter data were interpreted by two physicians specialized in clinical physiology (PL and AT).

## Statistical analyses

In the sample size calculation, we assumed that the true minimum HR was 10 bpm lower than the current reference value of 94 bpm for 4–10-day-old newborns [9]. Using a standard deviation (SD) for HR of 13 bpm, as reported by Southall et al. [9], a sample size calculation showed that 28 observations were needed (force .80, *P* = .05). To ensure a large enough sample size, 70 newborns were planned to recruit.

Age was calculated in hours from birth and converted into days. Means and SDs were calculated for normally distributed variables, and medians and ranges were calculated for skew-distributed variables. Frequencies and percentages were used for categorical variables. Pearson correlation was used whenever appropriate. Linear regression analysis was used in univariate and multivariate HR analyses. The age-specific limits, with -2 and 2 SDs, for HRs were calculated using linear regression analysis coefficients and residuals. In case of missing data, complete case analysis was used. The statistical significance level was defined as *P* (two-sided) < .05, and 95% confidence intervals (CIs) were presented whenever appropriate. Statistical analyses were performed using IBM SPSS Statistics for Macintosh version 28.0 (IBM, Armonk, NY, USA).

## Results

The study population consisted of 70 newborns (46% males) (Table 1). One infant had signs of ventricular hypertrophy on ECG. All other infants had normal ECG findings. Echo was performed on three infants due to a systolic murmur (n = 1), abnormal ECG (n = 1), and ventricular tachycardia (VT) in the Holter recording (n = 1), and they all had normal echo findings appropriate to their age.

**Table 1:** Study Population Characteristics (*N* = 70)

Characteristics	
Male, *n* (%)	32 (46)
Age in days, mean (SD)	6.4 (1.7)
Gestational age in weeks, median (range)	40 (37–42)
Birth weight, in grams, mean (SD)	3590 (375)
Birth weight between -2 and 2 SD, *n* (%)	70 (100)
1-min Apgar, median (range)	9 (7–9)
5-min Apgar, median (range)	9 (8–10)
Cord pH, mean (SD)	7.25 (0.07)^a^
Spontaneous vaginal delivery, *n* (%)	68 (97)

^a^Seven cases missing

The mean age at the start of the monitoring period was 6.4 days (range: 2.7–9.5 days) (Table 1). The means for the minimum and maximum HRs were 88 bpm and 208 bpm, respectively (Table 2). The minimum HR showed sinus rhythm in most cases (sinus rhythm, n = 68 [97%]; junctional rhythm, n = 2 [3%]). The minimum HR occurred during sleeping (n = 56 [80%]), eating (n = 4 [6%]), or unspecified activity (n = 10 [14%]). The maximum HR showed sinus rhythm in all cases. The maximum HR occurred during crying or agitation (n = 50 [71%]), eating (n = 6 [9%]), sleeping (n = 2 [3%]), hiccupping (n = 1 [1%]), or unspecified activity (n = 11 [16%]). No cardiac symptoms were reported during the recording.

**Table 2:** Holter Results

Parameters	
Minimum heart rate in bpm, mean (SD)	88 (12)
Mean heart rate in bpm, mean (SD)	141 (10)
Maximum heart rate in bpm, mean (SD)	208 (13)
Maximum R-R interval in seconds, mean (SD)	0.80 (0.12)
Recording duration in hours, median (range)	23 (18–25)
APCs, beats in total, *n* (%)	
≥ 1	54 (77)
1–10	39 (56)
11–100	14 (20)
101–1000	0 (0)
≥ 1000	1 (1)
VPCs, beats in total, *n* (%)	
≥ 1	28 (40)
1–10	24 (34)
11–100	2 (3)
101–1000	0 (0)
≥ 1000	2 (3)
Supraventricular tachycardia, *n* (%)	5 (7)
Ventricular tachycardia, *n* (%)	1 (1)

**Fig. 2:**
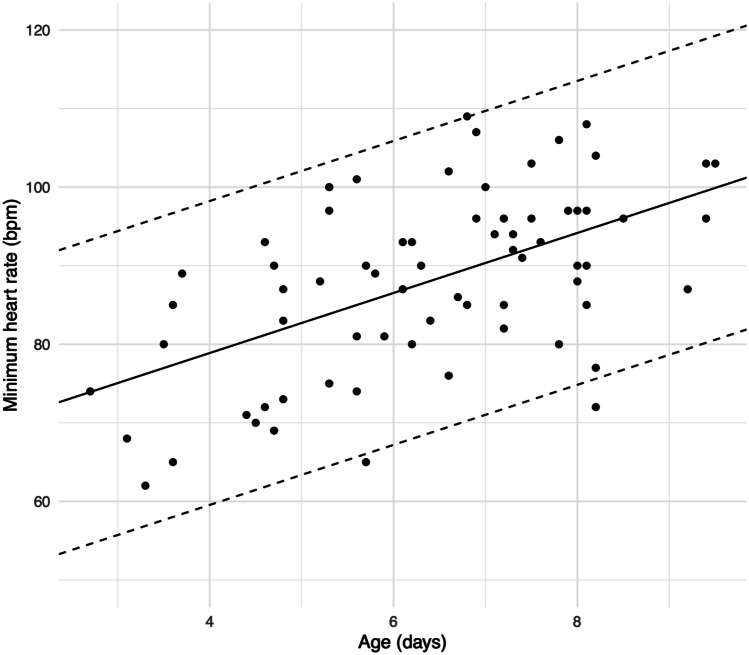
Association between minimum heart rate and age. The solid line represents the linear regression analysis curve between x and y. The dashed lines represent ± 2 standard deviations of linear regression analysis residuals

**Fig. 3:**
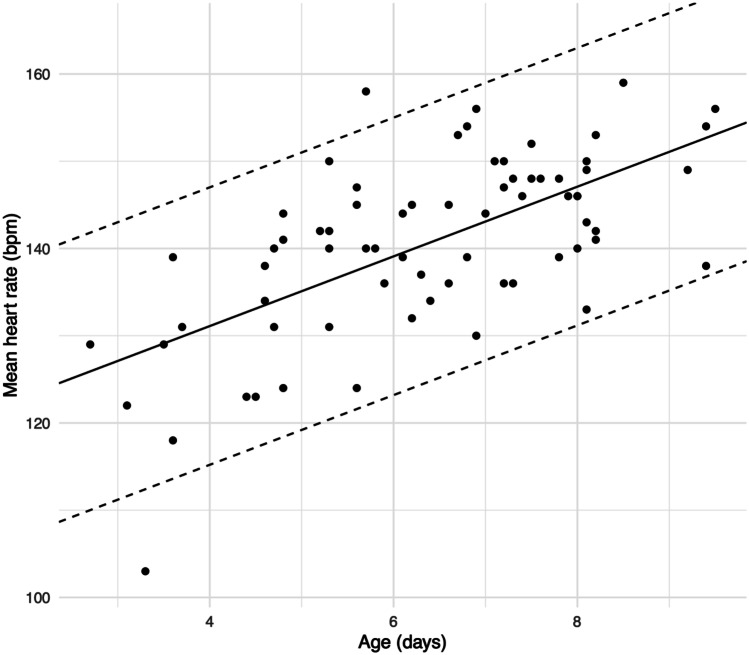
Association between mean heart rate and age. The solid line represents the linear regression analysis curve between x and y. The dashed lines represent ± 2 standard deviations of linear regression analysis residuals

**Fig. 4:**
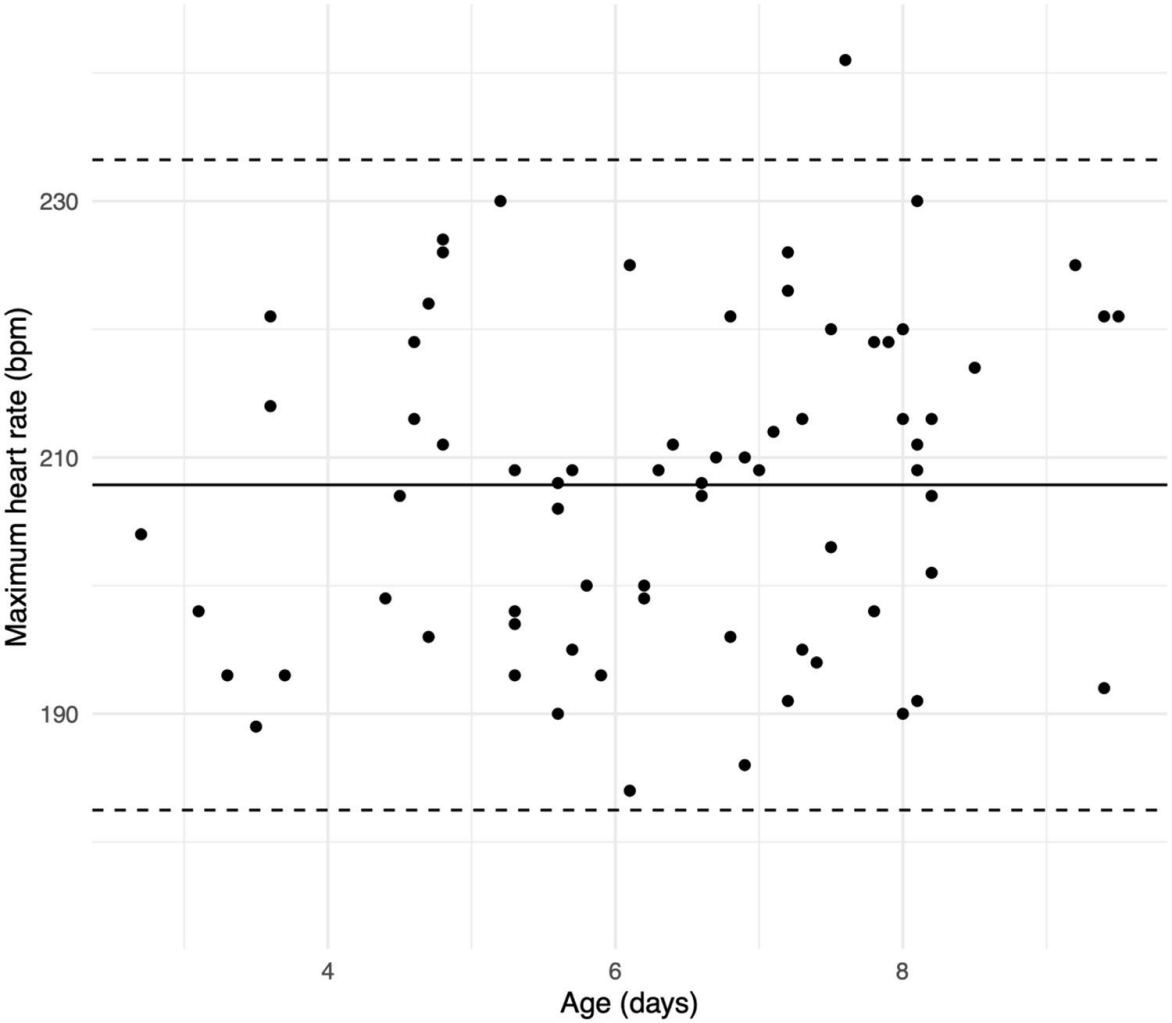
Association between maximum heart rate and age. The solid line represents the mean of y, and the dashed lines represent its ± 2 standard deviations

Positive correlations were observed between age and minimum HR (*r* = .55; *P* < .001) (Fig. 2) and age and mean HR (*r* = .64; *P* < .001) (Fig. 3). Age did not correlate with maximum HR (Fig. 4). According to the univariate linear regression analyses, each consecutive day of age raised the minimum and mean HRs by 3.8 bpm (95% CI: 2.4, 5.2; *P* < .001) and 4.0 bpm (95% CI: 2.8, 5.2; *P* < .001), respectively (Table 3). No statistically significant associations were found between sex and HR or gestational age (GA) at birth and HR in the univariate analyses.

**Table 3:** Linear Regression Analyses of Heart Rate

		Minimum heart rate, bpm		Mean heart rate, bpm		Maximum heart rate, bpm	
Model	Variables	*B* (95% CI)	*P*	*B* (95% CI)	*P*	*B* (95% CI)	*P*
1	(Constant)	63.6 (54.3, 72.9)	< .001	115.1 (107.4, 122.8)	< .001	198.7 (186.6, 210.7)	< .001
	Age, days	3.8 (2.4, 5.2)	< .001	4.0 (2.8, 5.2)	< .001	1.4 (-.4, 3.3)	.12
2	(Constant)	65.9 (56.4, 75.4)	< .001	116.0 (108.4, 123.7)	< .001	198.2 (185.8, 210.6)	< .001
	Age, days	3.8 (2.4, 5.2)	< .001	4.1 (3.0, 5.2)	< .001	1.6 (-.3, 3.4)	.09
	Male sex	-4.7 (-9.3, -.05)	< .05	-2.9 (-6.7, .8)	.12	-.5 (-6.6, 5.5)	.86
	GA, weeks	-.6 (-2.8, 1.6)	.59	-2.1 (-3.9, -.3)	.02	-2.3 (-5.1, .6)	.12

Multivariate linear regression analyses for HR were performed with the independent variables of age, sex, and GA (Table 3). According to these analyses, age and male sex had a statistically significant correlation with minimum HR (age coefficient: 3.8 bpm [95% CI: 2.4, 5.2]; *P* < .001; sex coefficient: -4.7 bpm [95% CI: -9.3, -.05]; *P* < .05), and age and GA had a statistically significant correlation with mean HR (age coefficient: 4.1 bpm [95% CI: 3.0, 5.2]; *P* < .001; GA coefficient: -2.1 bpm [95% CI: -3.9, -.3]; *P* = .02). The association between age and HR was not significantly affected after adding variables (sex and GA) to the linear regression model (Table 3). None of the variables (age, sex and GA) showed a statistically significant correlation with maximum HR in the multivariate linear regression analysis.

The age-specific limits for minimum HR were calculated using the following formula based on regression analysis results: 63.6 bpm + 3.8 * Age (days); ± 2 * 9.7 (Table 4). A similar formula was used for mean HR: 115.1 bpm + 4.0 * Age (days); ± 2 * 8.0. The lowest calculated limit for HR was 56 bpm for 3-day-old newborns and 78 bpm for 9-day-old newborns.

**Table 4:** Heart Rate Reference Values for Holter Monitoring

	Minimum heart rate, bpm^a^		Mean heart rate, bpm^a^		Maximum heart rate, bpm^b^	
Age, days	Lower limit	Upper limit	Lower limit	Upper limit	Lower limit	Upper limit
3	56	94	111	143	182	234
4	59	98	115	147	182	234
5	63	102	119	151	182	234
6	67	106	123	155	182	234
7	71	110	127	159	182	234
8	75	113	131	163	182	234
9	78	117	135	167	182	234

^a^Formulas for minimum and mean heart rates were generated from linear regression analysis with heart rate as the dependent variable and age as the independent variable. The formulas were written as follows: B(Constant) + B(Age) * Days; ± 2 * Residual Standard Deviation. Minimum heart rate = 63.6 bpm + 3.8 * Age (days); ± 2 * 9.7. Mean heart rate = 115.1 bpm + 4.0 * Age (days); ± 2 * 8.0

^b^Maximum heart rate = Mean ± 2 * Standard Deviation

One or more APCs and one or more VPCs were observed in 54 (77%) and 28 (40%) infants’ recordings, respectively (Table 2). The percentages for APCs and VPCs were ≤ 1% of total beats in all the recordings. Five neonates (7%) had one, two, or three 3-beat-long to 6-beat-long episodes of supraventricular tachycardia (SVT) with a median frequency of 193 bpm (range: 180–241 bpm). One infant (1%) had a 4-beat-long episode of VT with a frequency of 208 bpm. The total number of APCs or VPCs was low (range: 5–47 beats) in recordings with SVT or VT. All the infants with SVT or VT had normal results in the control recordings.

## Discussion

The present study showed an increase of 20 bpm in the minimum and mean HRs of healthy term newborns between the 3rd and 9th days of life. In addition, the proportion of infants with extrasystoles was higher than previously reported, and short SVTs and VTs were observed in 9% of the recordings in healthy newborns.

Increasing HR during the neonatal period has been reported in previous studies [10–14]. In these studies, HR measurements were seldom based on Holter recordings or other long-term HR monitoring. In a large review by Fleming et al., the median HR increased from 127 bpm at birth to 145 bpm at 1 month [10]. While this review included studies with various HR measurements (e.g., ﻿ECG, ﻿blood pressure monitors, ﻿manual measurements, ﻿echo, ﻿pulse oximeters), only four studies in the review were Holter studies on newborns. Richards et al. studied 110 term infants with sequential Holter recordings during the first 6 months of their lives [11]. In this study, the mean HR increased from 116 to 141 bpm in the first 15 days. However, the researchers only reported HR during periods of regular breathing, with a total duration of 3 h (mean) in the first 15 days.

Previous findings on increasing HR during the neonatal period have been observations of mean or median heart rate measurements. To our knowledge, the present study is the first to report increases in both minimum and mean HR with postnatal age. Inaccuracies in the diaries held by parents during the recording prevented us from separately evaluating waking and sleeping HRs. However, HR has previously been observed to increase at similar rates during both waking and sleeping states during the first month of life [12, 15]. The maximum HR in our study population was not age-dependent. Since no stress test was performed, we do not know whether the recorded maximum HR was the true maximum HR of the evaluated newborns.

Due to the postnatal cardiovascular stabilization period, we did not perform Holter monitoring on our subjects during the first couple of days of life. However, according to previous studies, HR progression is not linear from birth. In HR variability studies, HR variability increases and HR declines during the first postnatal days [1]. Makarov et al. reported differences in the waking mean HR between days 1, 2, and 4—with day 2’s HR being the lowest [13]. In another study, the median HR (measured by a blood pressure monitor) declined progressively in the first 3 days and increased on the 4th day [14]. Both the early decline and subsequent increase of HR have been explained by the postnatal acceleration of autonomic nervous system maturation [1, 12].

Previous Holter studies support our result that the minimum HR for newborns is lower than 80 bpm (Table 5). The mean values for HRs were somewhat similar in these studies. The small differences in the values between the studies can be explained by the small sample sizes. However, none of these studies considered the possible effect of age on HR. Our findings highlight the importance of more age-specific HR thresholds. If the HR thresholds were equal in all neonates, the youngest newborns would likely be misclassified as sick, potentially leading to prolonged hospitalization. Furthermore, the use of the same reference values for all newborns may cause false-negative findings in older newborns.

**Table 5:** Heart Rate Values in Holter Studies

			Minimum heart rate, bpm			Mean heart rate, bpm		
Study	*N*	Age, days	-2 SD	Mean	2 SD	-2 SD	Mean	2 SD
Present study	70	3–9	64	88	112	121	141	161
Makarov et al. [13]	20	1–4	57	85	113	101	135	169
Alpay et al. [24]	25	3–10	61	93	125	125	143	161
Nagashima et al. [21]	63	0–1	66	96	126			
Montague et al. [30]	29	1–6	70	92	114	110	130	150
Southall et al. [9]	134							
		1–3	69	93	117			
		4–10	68	94	120			

Both male sex and GA had negative correlations with HR if age was included in the regression analysis. Previous studies’ findings on gender differences in the HRs of newborns are controversial [16–18]. In the present study, gender difference was observed only at the minimum heart rate. An inverse correlation between GA and HR has been reported [5, 19]. In these studies, the effect of GA on HR persisted after adjustment for confounding factors, such as birth weight, postnatal age and sex. The direct effect of GA on HR can be explained by the maturation of the autonomic nervous system. Premature newborns have ﻿a predominance of sympathetic tone, and cardiac parasympathetic activity increases progressively with GA [1, 20].

The frequency of extrasystoles has varied significantly between previous Holter studies [13, 21, 22]. This is likely due to the studies’ dissimilar equipment, small sample sizes, and diverse age ranges. The frequencies of APCs and VPCs in our previous Holter study [23] and the present study were higher than previously reported. For instance, Southall et al. found APCs in 19/134 (14%) newborns aged under 10 days, but they counted extrasystoles in randomly selected hours of recording [9]. In a Holter study of 25 healthy newborns with a population similar to present study’s (age range: 3–10 days; mean age: 6.5 days), 8% of the infants had APCs, and 28% had VPCs [24]. We conducted both automated and manual analyses of extrasystoles to ensure the reliability of the results. Most infants had < 100 APCs or VPCs during the 24-h recording. In support of previous studies’ findings, we can conclude that the presence of a small amount of extrasystoles is common in healthy newborns. Further research is warranted to evaluate the upper limit for normal amounts of extrasystoles in newborns.

The estimated prevalence of SVT in infancy is between 1/250 and 1/1000 [25]. SVT in the first year of life is considered an arrhythmia that requires prophylactic antiarrhythmic treatment until the tendency for SVT ceases spontaneously [26]. Isolated VT in infants without a structural heart disease is often an incidental finding in the course of evaluating an unrelated condition, and most of these infants may be managed with conservative observation alone [27, 28].

Interestingly, 9% of our healthy subjects had short episodes of SVT or VT that disappeared during follow-up and without treatment. In a retrospective study of an institution’s pediatric SVT, 74% of infants younger than 1 year presented no symptoms at diagnosis, and in 44% of infants, the diagnosis was made during routine investigations or monitoring [29]. Because infants may be asymptomatic during brief SVT episodes, the incidence of infantile SVT is likely underestimated. The incidental short tachycardias in our healthy newborn population could present as a subtype of SVT in infancy. Further studies are needed to evaluate whether the tendency for short tachycardias ceases at younger age than the tendency for longer or sustained SVT. Our findings suggest that some newborns with short SVTs could be managed with close follow-up and without prophylactic antiarrhythmic treatment.

The strengths of this study include its homogenous study population of healthy newborns, careful exclusion of neonatal and maternal conditions that could potentially affect HR, thorough Holter data analysis, and, compared to previous Holter studies, its sample size. However, the study is also limited by its sample size due to the small number of cases in each age group.

## Conclusions

The present study showed clinically relevant age-dependent changes in minimum and mean HR by 9 days of age. Daily reference values for HR could be adopted in the interpretation of HR monitoring results in newborns. Larger and broader sample sizes could better verify the age-specific reference values and changes to these values with advancing age. A small number of extrasystoles are common in healthy newborns, and short isolated tachycardias may be normal in this age group.

## Data Availability

The data that support the findings of this study are available on request from the corresponding author.

## Abbreviations

APC: Atrial premature contraction
Bpm: Beats per minute
CI: Confidence interval
ECG: Electrocardiogram
Echo: Echocardiography
GA: Gestational age
HR: Heart rate
SD: Standard deviation
SVT: Supraventricular tachycardia
VPC: Ventricular premature contraction
VT: Ventricular tachycardia
